# Oncological similarities between large type 3 and type 4 tumors in patients with resectable gastric cancer: a propensity score-matched analysis of a multi-institutional dataset

**DOI:** 10.1007/s10120-024-01546-x

**Published:** 2024-08-22

**Authors:** Koki Nakanishi, Mitsuro Kanda, Seiji Ito, Yoshinari Mochizuki, Hitoshi Teramoto, Kiyoshi Ishigure, Toshifumi Murai, Takahiro Asada, Akiharu Ishiyama, Hidenobu Matsushita, Dai Shimizu, Chie Tanaka, Michitaka Fujiwara, Kenta Murotani, Yasuhiro Kodera

**Affiliations:** 1 Department of Gastroenterological Surgery, Nagoya University Graduate School of Medicine, 65 Tsurumai-Cho, Showa-Ku, Nagoya, 466-8550 Japan; 2 Department of Gastroenterological Surgery, Aichi Cancer Center, Nagoya, Japan; 3 Department of Surgery, Komaki City Hospital, Komaki, Japan; 4 Department of Surgery, Yokkaichi Municipal Hospital, Yokkaichi, Japan; 5 Department of Surgery, Konan Kosei Hospital, Konan, Japan; 6 Department of Surgery, Ichinomiya Municipal Hospital, Ichinomiya, Japan; 7 Department of Surgery, Gifu Prefectural Tajimi Hospital, Tajimi, Japan; 8 Department of Surgery, Okazaki City Hospital, Okazaki, Japan; 9 Department of Surgery, Tosei General Hospital, Seto, Japan; 10 Medical xR Center, Nagoya University Graduate School of Medicine, Nagoya, Japan; 11 Biostatistics Center, Graduate School of Medicine, Kurume University, Kurume, Japan

**Keywords:** Gastric cancer, Prognosis, Neoadjuvant therapy, Retrospective study, Propensity score

## Abstract

**Background:**

Large type 3 (diameter ≥ 8 cm) and type 4 gastric cancers have been arbitrarily combined in Japan as a single entity. However, whether these two types are oncologically similar remain unclear. This study aimed to clarify this issue.

**Methods:**

In this retrospective study, we analyzed a database of 3,575 patients from nine institutions who underwent gastrectomy between 2010 and 2014. Using propensity scores to balance significant variables, we compared prognoses and tumor recurrences.

**Results:**

Of patients with clinical T3/T4 who underwent R0 resection, 75 and 73 had large type 3 and 4 tumors, respectively. Patients with type 4 tumors had significantly lower overall survival rates than those of patients with large type 3 tumors (hazard ratio [HR] 1.77; 95% confidence interval [CI] 1.14–2.74). However, among the large type 3 tumors, a remarkable difference in prognosis was observed between the differentiated and undifferentiated histological types. A comparison was made between large type 3 with undifferentiated phenotype and type 4, each with 39 patients after propensity score matching. Outcomes in both groups were similar in terms of overall survival (HR 1.28; 95% CI 0.73–2.25) and relapse-free survival (HR 1.34; 95% CI 0.80–2.27). No statistically significant differences were observed in the incidence of peritoneal recurrence (35.9% vs. 46.1%, *P* = 0.36) and lymph node recurrence (25.6% vs. 12.8%, *P* = 0.15).

**Conclusions:**

Large type 3 tumors with undifferentiated phenotype and type 4 tumors were oncologically similar. This subgroup could be considered as a new entity for future clinical trials.

**Supplementary Information:**

The online version contains supplementary material available at 10.1007/s10120-024-01546-x.

## Introduction

Macroscopic types of gastric cancer have been defined by the Japanese Gastric Cancer Association (JGCA) guidelines [1], and are one of several items to be recorded in the database as a prognostic determinant [2, 3], among other prognostic factors such as the number of metastatic nodes, depth of invasion, tumor size, microscopic lymphatic invasion, and histological type [4–8]. The macroscopic type is correlated with the histological type, depth of invasion, tumor growth pattern, and molecular malignant features [9]. According to the nationwide registry of the JGCA, patients with type 4 tumors have a particularly poor prognosis among patients with resectable gastric cancer, followed by those with type 3 tumors [8].

Among type 3 tumors, those with a maximum diameter ≥ 8 cm are termed “large type 3”. Sasako et al. proposed the addition of these tumors to type 4 to establish a new entity of gastric cancer with a dismal prognosis and to prove the benefits of a neoadjuvant strategy with this population in a clinical trial by the Japan Clinical Oncology Group (JCOG) [10]. Their opinion was that accrual of only type 4 tumors would be highly time-consuming. This proposal was based on the observation that large type 3 tumors exhibit aggressive biological behavior similar to that of type 4 tumors [11, 12]. In due course, neoadjuvant chemotherapy with S-1 + cisplatin was delivered to patients with resectable large type 3 and type 4 tumors (JCOG0210) and was found to be a promising strategy [12]. However, no survival benefit of neoadjuvant S-1 + cisplatin was observed in a subsequent phase III trial (JCOG0501) [13], and more intensive triplet regimens are currently being tested [14].

Currently, there is some skepticism regarding treating the large type 3 tumors as an equivalent of type 4. The JCOG0210 trial presented survival curves for each macroscopic type and reported a trend of shorter overall survival (OS) in patients with large type 3 tumors, although the differences were not significant [12]. The JCOG0501 trial did not provide survival information based on the macroscopic tumor type. In contrast, a retrospective observational study in Korea reported that neoadjuvant chemotherapy improved the prognosis of patients with large type 3 tumors but not of those with type 4 tumors [15]. Neoadjuvant chemotherapy was found to be unfavorable for patients with signet ring cell carcinoma, which is strongly associated with type 4 tumors [16]. In fact, only patients with type 4 tumors are eligible for the ongoing development of perioperative intraperitoneal/systemic chemotherapy that focuses on the avoidance of peritoneal recurrences [17].

Thus, the issue of similarity or disparity between the large type 3 and type 4 tumors needs to be clarified for designing future clinical trials. This study aimed to address this issue using a multi-institutional retrospective dataset.

## Patients and methods

### Patient selection

We retrospectively reviewed the clinical data of 3,575 patients who underwent gastrectomy for gastric cancer between January 2010 and December 2014. The data were acquired from the medical records of nine institutions. To guarantee the quality of surgery, we selected institutions that performed more than 50 gastrectomies for gastric cancer each year. Board-certified surgeons from the Japanese Society of Gastroenterological Surgery performed or supervised all surgeries. Eligible patients were selected for analysis based on the following criteria: pathologically proven gastric adenocarcinoma, macroscopic large type 3 (≥ 8 cm diameter) or type 4 tumors, clinical T3 or T4 tumors, no distant metastasis, R0 resection, and sufficient data for analysis. Patients with other concurrent cancers were excluded.

### Surgery and patient management

Clinical stages were classified based on the 14th edition of the Japanese Classification of Gastric Carcinoma [18]. Severe comorbidities were defined as diseases classified as American Society of Anesthesiologists Physical Status 3, such as poorly controlled diabetes, poorly controlled hypertension, chronic obstructive pulmonary disease, severe obesity (body mass index ≥ 40), active hepatitis, history of myocardial infarction, pacemaker implantation, and dialysis. Other clinical and histopathological factors were recorded according to the 15th edition of the Japanese Classification of Gastric Carcinoma [1], in which tubular and papillary adenocarcinomas are defined as differentiated adenocarcinomas, whereas poorly differentiated adenocarcinomas, signet-ring cell carcinomas, and mucinous adenocarcinomas are defined as undifferentiated adenocarcinomas. Multidisciplinary conferences were held primarily to evaluate patients with stage III disease, and neoadjuvant chemotherapy was administered at the physician’s discretion, as indicated in the protocol of clinical trials at the time. We analyzed data on postoperative complications occurring within 30 days after surgery according to the Clavien–Dindo classification system [19, 20], even when patients were treated or followed at an outpatient clinic. Postoperative adjuvant S-1 monotherapy was recommended for all patients with pathological stage II/III disease unless contraindicated by the patient’s condition, except for those with T1 or T3N0 gastric cancers. Patient follow-up was performed according to the Japanese Gastric Cancer Treatment guidelines [21].

### Propensity score-matched analysis

We used propensity score matching to balance the significant variables between the two patient groups that underwent comparison. Propensity scores were estimated using a logistic regression model based on sex, age, presence of severe comorbidities, neoadjuvant chemotherapy (none or received), clinical N stage (N0, N1, N2, or N3), gastrectomy type (total gastrectomy or other), extent of lymphadenectomy (≥ D2 or < D2), pathological stage (stage II or stage III), and postoperative adjuvant chemotherapy (none or received). One-to-one matching without replacement was performed using a 0.1 caliper width, and the resulting score-matched pairs were used in the subsequent analyses.

### Ethics

This study protocol was in accordance with the principles of the Declaration of Helsinki and was approved by the institutional ethical review board of Nagoya University Graduate School of Medicine (No. 2017–0104). Written informed consent for treatment was obtained from all the patients. We used an opt-out recruitment strategy in accordance with Japanese government policy because we exclusively analyzed retrospective clinical data without any intervention by the investigators. Patients were excluded if they were unwilling to participate in the study.

## Statistical analysis

Correlations between each group and clinicopathological variables were analyzed using the Chi-square test or Fisher’s exact test for categorical variables and the Mann–Whitney U test for continuous variables. Survival rates were estimated using the Kaplan–Meier method, and the overall differences between survival curves were compared using the Cox proportional hazards model. OS was defined as the time from initial therapeutic treatment (gastrectomy or neoadjuvant chemotherapy) to all-cause death or the date of the last follow-up. Relapse-free survival (RFS) was defined as the period between the day of initial therapeutic treatment and the time of disease recurrence. Data were analyzed using the JMP version 16 software (JMP, SAS Institute, Cary, NC, USA). Statistical significance was set at* P* < 0.05.

## Results

### Patients’ characteristics of full cohort analysis

**Fig. 1:**
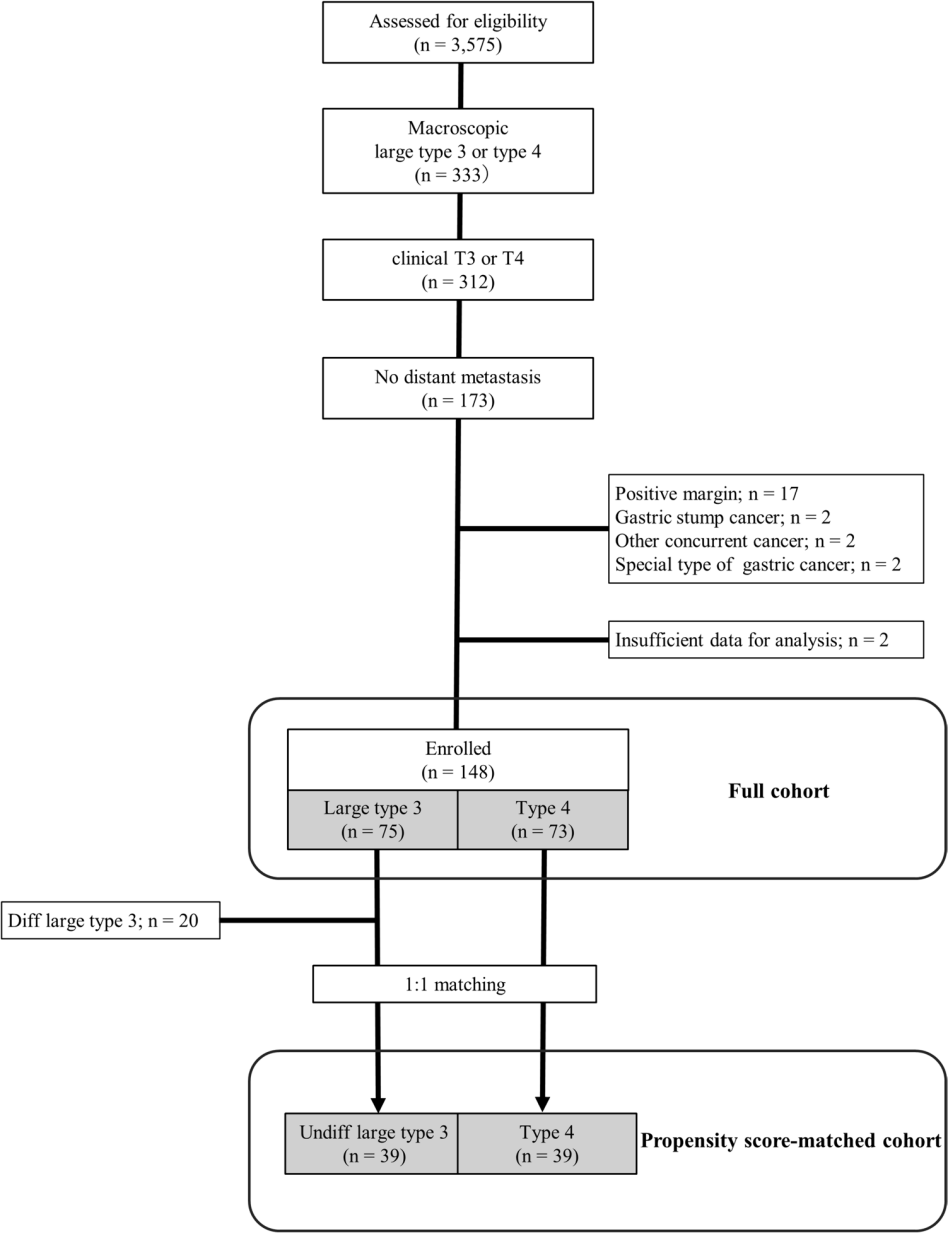
Flowchart of patient enrolment. Diff, differentiated; Undiff, undifferentiated

A total of 148 patients with either large type 3 (*n* = 75) or type 4 (*n* = 73) tumors met the eligibility criteria and underwent subsequent analyses. Figure 1 presents a CONSORT flowchart. Table 1 shows the demographic characteristics of each macroscopic tumor type. Patients in the group with type 4 tumors were significantly younger than those with large type 3 tumors (mean age: 67.5 vs. 71.9, *P* = 0.02), with a higher proportion of patients with the undifferentiated histological tumor type (88% vs. 73%, *P* = 0.03). The group of patients with type 4 tumors tended to have a higher rate of total gastrectomy and a more advanced pathological disease stage than those with large type 3 tumors, with no statistically significant difference.

**Table 1:** Patients’ characteristics categorized by macroscopic tumor types

	Full cohort				Propensity score-matched cohort			
	Large type 3 (*n* = 75)	Type 4 (*n* = 73)	*P* value	Standardized difference	Undifferentiated large typem3 (*n* = 39)	Type 4 (*n* = 39)	*P* value	Standardized difference
Sex			0.33	0.161			0.47	0.165
Male	49 (65%)	42 (58%)			25 (64%)	28 (72%)		
Female	26 (35%)	31 (42%)			14 (36%)	11 (28%)		
Age, years								
Mean ± SD	71.9 ± 10.2	67.5 ± 13.0	0.02	0.373	69.4 ± 11.3	70.6 ± 10.2	0.62	0.111
Severe comorbidities			0.89	0.022			0.59	0.122
Present	14 (19%)	13 (18%)			8 (21%)	10 (26%)		
Absent	61 (81%)	60 (82%)			31 (79%)	29 (74%)		
ECOG-PS			0.64	0.077			0.59	0.314
0	55 (73%)	51 (70%)			32 (82%)	29 (74%)		
≥ 1	20 (27%)	22 (30%)			7 (18%)	10 (26%)		
Tumor location			0.13	0.400			0.19	0.510
Upper	19 (25%)	17 (23%)				9 (23%)	10 (26%)	
Middle	14 (19%)	20 (27%)				7 (18%)	12 (31%)	
Lower	26 (35%)	14 (19%)				14 (36%)	6 (15%)	
Whole	16 (21%)	22 (30%)				9 (23%)	11 (28%)	
Tumor size, mm								
Mean ± SD	99 ± 21	96 ± 37	0.60	0.086	99 ± 42	96 ± 42	0.57	0.466
Histologic type			0.03	0.368				0.603
Differentiated	20 (27%)	9 (12%)			–	6 (15%)		
Undifferentiated	55 (73%)	64 (88%)			39 (100%)	33 (85%)		
Signet-ring cell component			0.17	0.227			0.65	0.104
Present	18 (24%)	25 (34%)			16 (41%)	18 (46%)		
Absent	57 (76%)	48 (66%)			23 (59%)	21 (54%)		
cStage (JGCA 14th)			0.12	0.457			0.79	0.313
IIA	5 (7%)	10 (14%)			4 (10%)	3 (8%)		
IIB	14 (19%)	24 (33%)			9 (23%)	12 (31%)		
IIIA	19 (25%)	14 (19%)			11 (28%)	8 (21%)		
IIIB	24 (32%)	16 (22%)			7 (18%)	10 (26%)		
IIIC	13 (17%)	9 (12%)			8 (21%)	6 (15%)		
Neoadjuvant chemotherapy			0.59	0.089			1.00	0
Received	16 (21%)	13 (18%)			9 (23%)	9 (23%)		
Not received	59 (79%)	60 (81%)			30 (77%)	30 (77%)		
Type of gastrectomy			0.06	0.314			0.61	0.114
Partial gastrectomy	27 (36%)	16 (22%)			12 (31%)	10 (26%)		
Total gastrectomy	48 (64%)	57 (78%)			27 (69%)	29 (74%)		
Lymph node dissection			0.77	0.022			0.56	0.134
D1/D1 +	14 (19%)	15 (21%)			8 (21%)	6 (15%)		
≥ D2	61 (81%)	58 (79%)			31 (79%)	33 (85%)		
pStage (JGCA 15th)			0.08	0.483			0.24	0.369
IIA	11 (15%)	2 (3%)			3 (8%)	1 (3%)		
IIB	9 (12%)	12 (16%)			5 (13%)	8 (21%)		
IIIA	20 (27%)	20 (27%)			9 (23%)	8 (21%)		
IIIB	17 (23%)	24 (33%)			10 (26%)	13 (33%)		
IIIC	18 (24%)	15 (21%)			12 (31%)	9 (23%)		
Adjuvant chemotherapy			0.44	0.099			0.47	0.224
Received	49 (65%)	52 (71%)			28 (72%)	25 (64%)		
Not received	26 (35%)	21 (29%)			11 (28%)	14 (36%)		

*SD*, standard deviation; *ECOG-PS*, eastern cooperative oncology group performance status; *JGCA*, Japanese gastric cancer association

### Survival and disease recurrence patterns of full cohort analysis

**Fig. 2:**
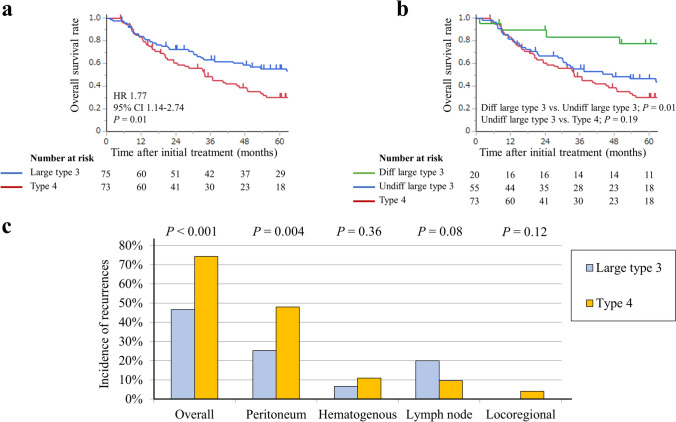
Survival and disease recurrence patterns in the full cohort analysis: **a** overall survival **b** relapse-free survival **c** Frequencies of sites of initial recurrence in each group. *HR*, hazard ratio; *CI*, confidence interval; Undiff, undifferentiated

Figure 2 shows the survival and disease recurrence patterns of the patients with each macroscopic tumor type. OS was significantly shorter in the type 4 group (hazard ratio [HR] 1.77; 95% confidence interval [CI] 1.14–2.74; *P* = 0.01) (Fig. 2a). Among the large type 3 tumors, a significant difference in OS was observed between patients with differentiated and undifferentiated histologic types (HR 4.63; 95% CI 1.41–15.1; *P* = 0.01), while minimal difference in OS was observed between patients with large type 3 with undifferentiated phenotype (undifferentiated large type 3) and all type 4 tumors (HR, 1.34; 95% CI 0.85–2.10; *P* = 0.19) (Fig. 2b). Patient characteristics of large type 3 tumors according to their histologic phenotypes are presented in Online Resource 2.

We investigated the patterns of initial disease recurrence after surgery and found a significantly higher prevalence of overall disease recurrence in patients with type 4 tumors than in those with large type 3 tumors (*P* < 0.001) (Fig. 2c). The group with type 4 tumors had a significantly higher rate of peritoneal recurrence than that in the group with large type 3 tumors (48.0% vs. 25.3%, *P* = 0.004), whereas the group with large type 3 tumors showed a trend toward a higher frequency of lymph node tumor recurrence than that in the group with type 4 tumors, although the difference was not statistically significant (20.0% vs. 9.5%, *P* = 0.08).

### Propensity score-matched analysis of patients’ characteristics

Based on the results of the full cohort analysis, we restricted the analysis to undifferentiated large type 3 and type 4 tumors to investigate whether they had oncological similarities even after adjusting for background factors using propensity-score matching. After one-to-one matching, 39 patients were included in both groups. The two groups of patients were well-balanced in terms of age, neoadjuvant chemotherapy, gastrectomy type, pathological stage, and postoperative adjuvant chemotherapy (Table 1).

### Propensity score-matched analysis of survival and disease recurrence patterns

**Fig. 3:**
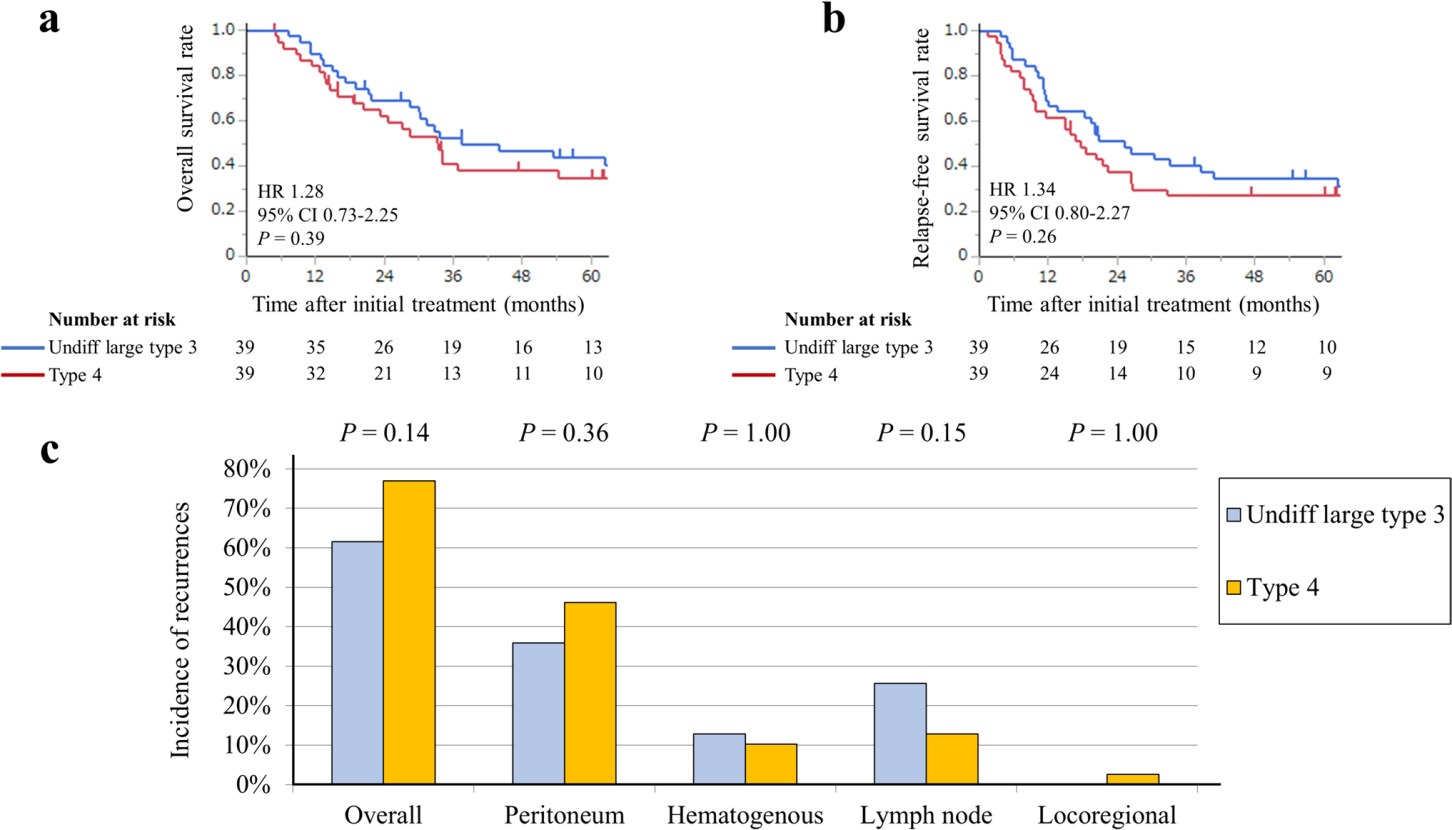
Survival and disease recurrence patterns in the propensity score-matched analysis: **a** overall survival **b** relapse-free survival **c** Frequencies of sites of initial recurrence in each group. HR, hazard ratio; CI, confidence interval; Undiff, undifferentiated

After propensity score matching, the outcomes of the two groups were similar in terms of both OS (HR 1.28; 95% CI 0.73–2.25; *P* = 0.39) (Fig. 3a) and RFS (HR 1.34; 95% CI 0.80–2.27; *P* = 0.26) (Fig. 3b). No statistically significant differences were observed between patients with undifferentiated large types 3 and 4 tumors in terms of peritoneal recurrence (35.9% vs. 46.1%, *P* = 0.36) and lymph node recurrence (25.6% vs. 12.8%, *P* = 0.15) (Fig. 3c).

### Chemotherapy and surgical outcomes

Detailed information regarding the perioperative chemotherapy in both groups of patients is presented in Table 2. Neoadjuvant chemotherapy was administered to nine patients (23%) in each group. In the group with undifferentiated large type 3 tumors, the regimens included S-1 + cisplatin (seven cases), S-1 + docetaxel + cisplatin (one case), and paclitaxel + cisplatin (one case), and all patients in the group with type 4 tumors received the S-1 + cisplatin regimen. The pathological responses were slightly less effective in the group with undifferentiated large type 3 tumors (grade 0, one case; grade 1a, two cases). Postoperative adjuvant chemotherapy was administered to 28 patients (72%) in the group with undifferentiated large type 3 tumors and to 25 patients (64%) in the group with type 4 tumors (*P* = 0.47). Almost all patients received S-1 monotherapy, even those with pStage III disease, based on the JGCA guidelines at that time.

**Table 2:** Perioperative chemotherapy courses

	Undifferentiated large type 3 (*n* = 39)	Type 4 (*n* = 39)
Neoadjuvant chemotherapy		
S-1 + cisplatin	7	9
S-1 + docetaxel + cisplatin	1	0
Paclitaxel + cisplatin	1	0
Pathological response		
0	1	0
1a	2	1
1b	2	2
2	2	4
Unknown	2	2
Adjuvant chemotherapy		
S-1	27	22
S-1 + docetaxel	1	1
S-1 + cisplatin	0	1
Capecitabine + Oxaliplatin	0	1

Detailed surgical outcomes of the two groups of patients are summarized in Table 3. All gastrectomy procedures were performed using an open approach. Total gastrectomy was performed in 27 patients (69%) in the group with undifferentiated large type 3 tumors and in 29 patients (74%) in the group with type 4 tumors. There were no significant differences between these groups in terms of the number of dissected lymph nodes or splenectomies. The group with undifferentiated large type 3 tumors had significantly longer operative times than those in the group with type 4 tumors (305 ± 65 vs. 280 ± 62 min; *P* = 0.04). The group with undifferentiated large type 3 tumors had trends of a higher frequency of Clavien–Dindo grade II or higher postoperative complications (47% vs. 26%, *P* = 0.06) and longer postoperative hospital stay (22 ± 9 days vs. 17 ± 9 days, *P* = 0.08) than that in the group with type 4 tumors, with no statistical significance.

**Table 3:** Patients’ surgical outcomes

	Undifferentiated large type 3 (*n* = 39)	Type 4 (*n* = 39)	*P* value
Approach			1.00
Open	39 (100%)	39 (100%)	
Operation time, min			
Mean ± SD	305 ± 65	280 ± 62	0.04
Intraoperative blood loss, ml			
Mean ± SD	534 ± 67	514 ± 67	0.83
Intraoperative blood transfusion	6 (15%)	3 (8%)	0.48
Dissected lymph node			
Mean ± SD	41 ± 16	43 ± 15	0.61
Splenectomy	15 (38%)	16 (41%)	0.82
Hospital stays after surgery, days			
Mean ± SD	22 ± 9	17 ± 9	0.08
Postoperative complications*			
II	9 (23%)	6 (15%)	
IIIa	8 (21%)	2 (5%)	
IIIb	1 (3%)	2 (5%)	
IV	0 (0%)	0 (0%)	
V	0 (0%)	0 (0%)	
Total	18 (47%)	10 (26%)	0.06
Reoperation	2 (5%)	2 (5%)	1.00
Readmission within 90 days	3 (8%)	5 (13%)	0.71

*SD* standard deviation

*According to the Clavien–Dindo classification

### Subgroup analysis

**Fig. 4:**
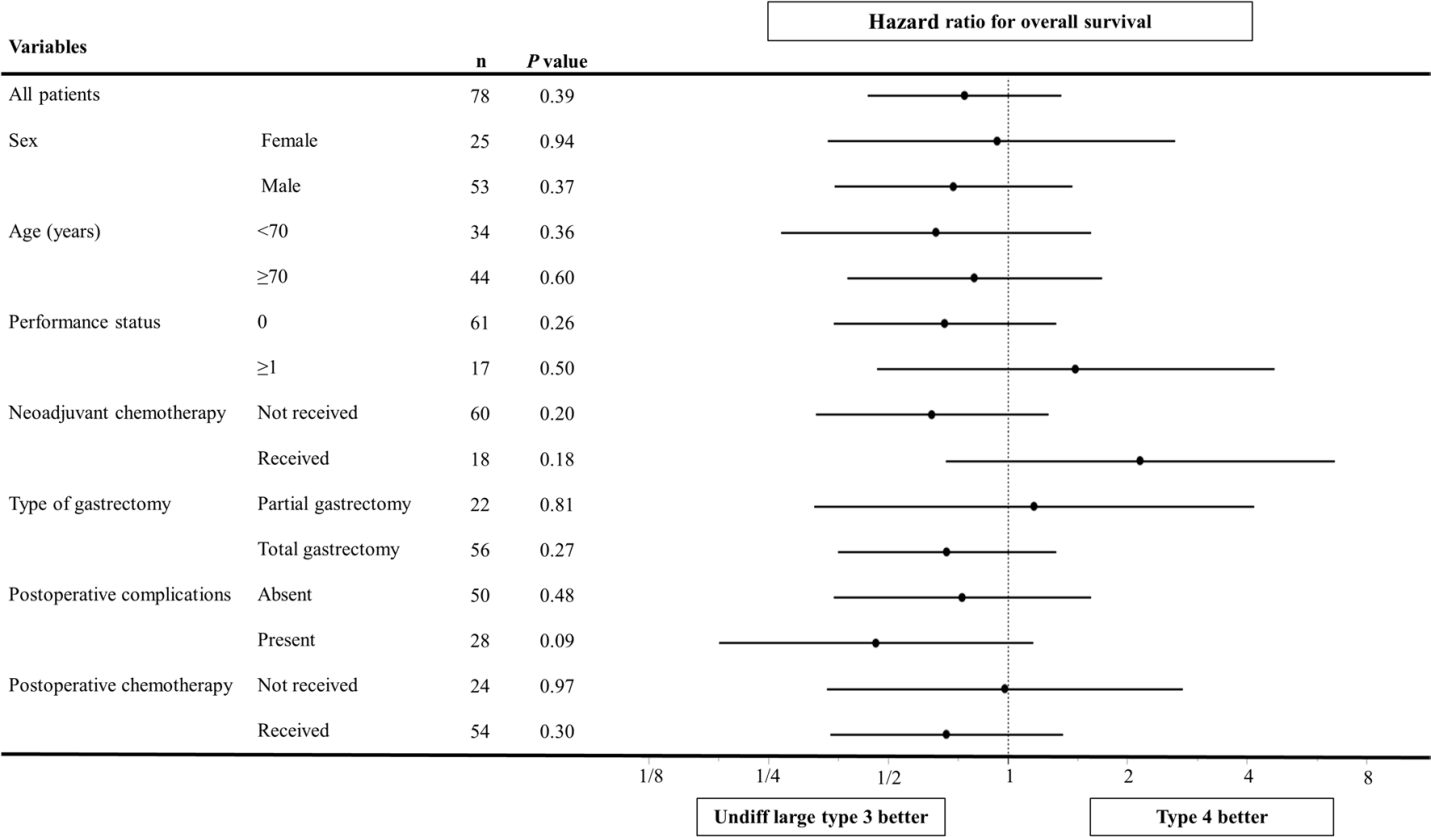
Forest plot evaluating differences in overall survival times between patients with undifferentiated large types 3 and 4 tumors. Undiff, undifferentiated

Subgroup analyses were conducted to compare further differences in prognoses between the two groups of patients. A forest plot demonstrated that in almost all subgroups, the group with type 4 tumors showed a trend towards shorter OS time than that in the group with undifferentiated large type 3 tumors (Fig. 4). In contrast, among patients who received neoadjuvant chemotherapy, the group with undifferentiated large type 3 tumors exhibited a trend of a shorter OS than that in the group with type 4 tumors (HR 2.15; 95% CI 0.69–6.64; *P* = 0.18).

The results for patients with undifferentiated large type 3 (*n* = 20) and type 4 (*n* = 15) tumors who received postoperative S-1 adjuvant chemotherapy without neoadjuvant chemotherapy are shown in Online Resource 1. The group with type 4 tumors showed trends of shorter OS (HR 1.73; 95% CI 0.71–4.23; *P* = 0.23) and progression-free survival (PFS) (HR 1.67; 95% CI 0.72–3.88; *P* = 0.23) than those in the group with undifferentiated large type 3 tumors. The group with type 4 tumors showed a higher frequency of peritoneal tumor recurrence trend than that in the group with undifferentiated large type 3 tumors (60.0% vs. 40.0%, *P* = 0.08).

## Discussion

Using a multi-institutional retrospective dataset, we investigated the oncological similarities between large types 3 and 4 tumors. Significant differences in patient age, prognosis, and initial recurrence patterns were observed between the two groups of patients. However, these oncological outcomes were similar when the comparisons were made between large type 3 tumors with undifferentiated phenotype and type 4 tumors.

Type 4 tumors have the poorest prognosis among all macroscopic tumor types [6] and are closely associated with peritoneal dissemination [22]. Type 3 tumors are the most common macroscopic type of resectable advanced gastric cancer [6], and large type 3 tumors often present with peritoneal dissemination and positive lavage cytology [23, 24]. Clinical trials have been conducted to develop treatments targeting these high-risk macroscopic cancer types [12, 13, 25, 26], and challenges persist in developing intensive perioperative chemotherapy regimens [14]. The efficacy of perioperative chemotherapy reportedly differs based on macroscopic or histological types [15, 16]; however, concerns have been raised as to whether it is appropriate to develop such a treatment strategy with the conventional target population consisting of large type 3 and type 4 tumors. Our study showed significant differences in prognosis and disease recurrence patterns between the large type 3 and type 4 tumors in a full cohort analysis. Type 4 tumors were associated with a shorter OS and a higher peritoneal disease recurrence rate, whereas large type 3 tumors had a higher lymph node disease recurrence rate. These results suggest that large type 3 and 4 tumors may have different oncological characteristics and mechanisms of progression. We observed a significant difference in prognosis between the differentiated and undifferentiated histological types in the large type 3 tumors. Therefore, we designated large type 3 tumors with undifferentiated phenotype and type 4 tumors as high-risk macroscopic types and conducted further analyses of survival and disease recurrence patterns. After adjusting for the patient background and treatment course, we concluded that there was an oncological similarity between large type 3 with undifferentiated phenotype and type 4 tumors, although some differences remained.

We found that differentiated large type 3 tumors have a notably better prognosis. The reason for this improved prognosis remains unclear. Given the rarity of differentiated large type 3 tumors and limited literature on this subgroup, we did not explore the underlying factors contributing to this outcome. However, based on our findings, we suggest that differentiated large type 3 tumors should not be grouped with type 4 tumors as a novel treatment strategy.

The macroscopic types of gastric cancer are defined by the JGCA guidelines and are considered one of the essential factors to be recorded. In addition, the discrimination between macroscopic types is not always an easy task. For example, some type 4 tumors are difficult to distinguish from the superficial tumor type, whereas others are classified as type 5 tumors. Similarly, large type 3 ulcers range from large ulcers (similar to type 2 ulcers) to diffuse infiltration with central ulcers (similar to type 4). In this study, a multi-institutional retrospective analysis was conducted, and the macroscopic type classification was performed at each institution without a central review. However, a central review of the macroscopic types is not routinely performed, even in prospective clinical trials. Large type 3 tumors with undifferentiated phenotype can be objectively classified based on the information obtained before treatment. In an attempt to identify effective treatments using large type 3 tumors and type 4 tumors as targets, it may be possible to eliminate bias by including histology in the eligibility criteria for clinical trials so that large type 3 tumors with differentiated phenotype and significantly more favorable outcomes can be excluded.

Surgery followed by postoperative adjuvant chemotherapy with S-1 monotherapy for 1 year was the standard treatment for the patient population included in this study. In addition, some patients have undergone neoadjuvant chemotherapy as part of clinical trials or practice. A subgroup analysis revealed that patients with undifferentiated large type 3 tumors had shorter OS than that of those with type 4 tumors among patients who received neoadjuvant chemotherapy, which is consistent with the findings of the JCOG0210 trial [12]. We found that the proportion of patients with a poor pathological response (grade 0, 1a) to neoadjuvant chemotherapy was higher in the group with undifferentiated large type 3 tumors than that in the group with type 4 tumors. The difference in response to neoadjuvant chemotherapy between the two groups of patients may have influenced the prognosis. Our results suggest the need for even more intensive neoadjuvant treatment to target these high-risk macroscopic cancers.

Surgical outcomes showed significant differences, with longer operative times and more postoperative complications in the group with undifferentiated large type 3 tumors than in those with type 4 tumors. The differences in tumor shrinkage due to neoadjuvant chemotherapy might have influenced the difficulty of surgery because patient characteristics, tumor size, and gastrectomy type were well balanced by propensity-score matching. However, all surgeries in our study were performed using open gastrectomies, and it is unclear whether the results would be similar in the era of minimally invasive surgery.

Our study included a relatively high number of differentiated type 4 tumors (12%), which increased to 15% after propensity score matching. This might be influenced by the multi-institutional nature of the study and the lack of a central review of macroscopic classification. Currently, there is no conclusive evidence regarding the impact of differences in histological phenotypes on the prognosis of patients with type 4 tumors. To address this, we stratified patients after propensity score matching into three groups, namely, differentiated type 4, undifferentiated type 4, and undifferentiated large type 3, and analyzed their survival outcomes. The survival curves did not show a significant difference between the differentiated and undifferentiated type 4 groups (Online Resource 3), suggesting that the high ratio of the differentiated phenotype in type 4 did not significantly affect the overall outcomes.

Our study had some limitations. First, although we performed propensity-score matching to eliminate the effects of confounding factors, differences in perioperative treatment between the two groups of patients could not be eliminated completely. Moreover, propensity-score matching reduced the sample size in both groups. Second, molecular analyses, which could provide new insights into the optimization of treatment strategies and improvement of patient outcomes, were not performed. Third, the current standard postoperative adjuvant chemotherapy for stage III gastric cancer is S-1 +  docetaxel, which was not delivered in a majority of patients in the current study. Fourth, owing to insufficient information on the histological types at preoperative biopsy, the histological types obtained from the resected specimens were used for analysis. Fifth, the classification of large type 3 tumors is unique to the Japanese study group and may not be well understood, particularly in Western countries. Since this study aimed to determine whether large type 3 and type 4 gastric cancers should be treated as a single group and not redefine the criteria for large type 3 tumors, establishing a cut-off value for large type 3 tumors was beyond the scope of this study. Future research should investigate and validate the appropriate cut-off value for large type 3 tumors to ensure the applicability of our findings across different clinical settings. Finally, resectable large type 3 and 4 gastric cancer tumors are rare and account for approximately 5% of all resectable gastric cancers. Although their specific natures may limit its usefulness in clinical practice, the analysis of large-scale multi-institutional datasets is considered valuable.

In conclusion, large type 3 tumors with undifferentiated phenotype and type 4 tumors have oncological similarities in patients with resectable gastric cancer. When developing the same novel treatment strategy for type 4 and large type 3 tumors, avoiding large type 3 tumors with differentiated phenotype may lead to less-biased results.

## Supplementary Information

Below is the link to the electronic supplementary material.

Supplementary Survival and disease recurrence patterns in propensity score-matched analysis in patients who received postoperative S-1 adjuvant chemotherapy without neoadjuvant chemotherapy (a) Overall survival (b) Relapse-free survival (c) Frequencies of sites of initial recurrence in each group. HR, hazard ratio; CI, confidence interval; Undiff, undifferentiated (TIF 884 KB)

Supplementary Patients' characteristics with large type 3 tumor by histologic phenotypes (TIF 865 KB)

Supplementary file3 (DOCX 25 KB)
