# Supplementary figures and images for: Oncological similarities between large type 3 and type 4 tumors in patients with resectable gastric cancer: a propensity score-matched analysis of a multi-institutional dataset
Source: Gastric Cancer. 2024 Aug 22;27(6):1331–41. doi: 10.1007/s10120-024-01546-x (PMC11513756; doi:10.1007/s10120-024-01546-x)

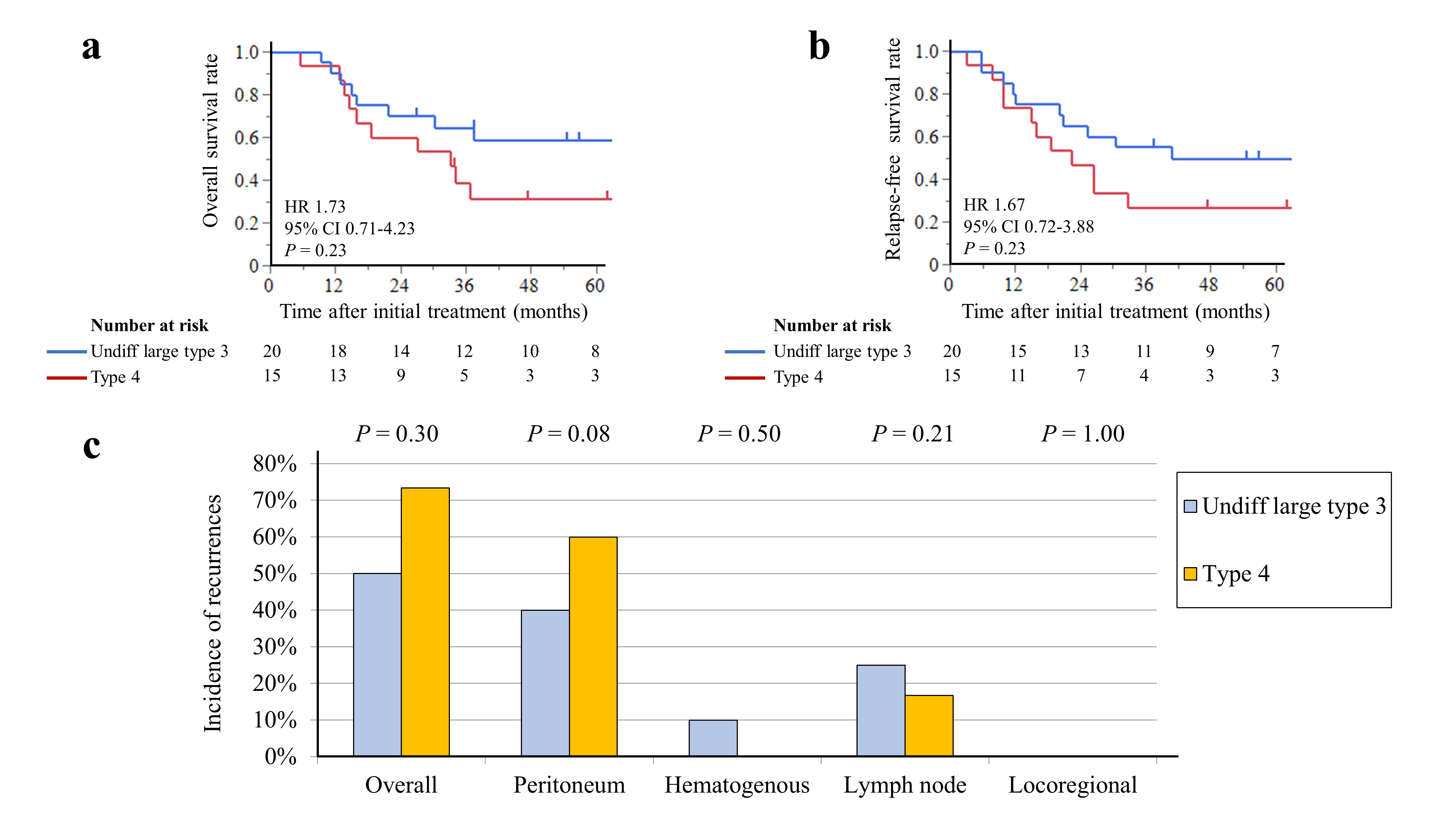

Supplement: Supplementary file 1 — Supplementary Survival and disease recurrence patterns in propensity score-matched analysis in patients who received postoperative S-1 adjuvant chemotherapy without neoadjuvant chemotherapy (a) Overall survival (b) Relapse-free survival (c) Frequencies of sites of initial recurrence in each group. HR, hazard ratio; CI, confidence interval; Undiff, undifferentiated (TIF 884 KB) [file 10120_2024_1546_MOESM1_ESM.tif]

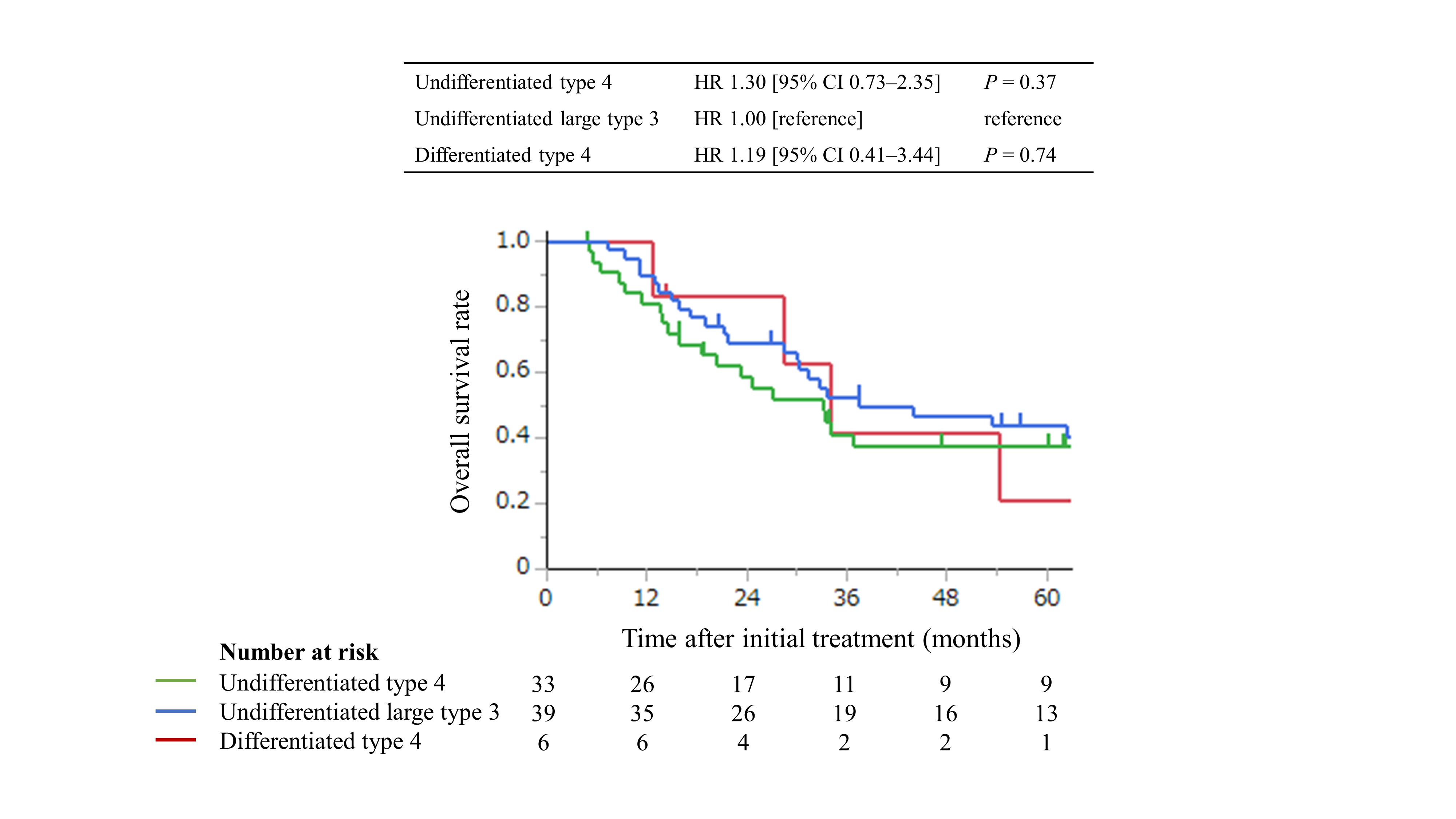

Supplement: Supplementary file 2 — Supplementary Patients' characteristics with large type 3 tumor by histologic phenotypes (TIF 865 KB) [file 10120_2024_1546_MOESM2_ESM.tif]
