# Supplementary material for: Oncological similarities between large type 3 and type 4 tumors in patients with resectable gastric cancer: a propensity score-matched analysis of a multi-institutional dataset
Source: Gastric Cancer. 2024 Aug 22;27(6):1331–41. doi: 10.1007/s10120-024-01546-x (PMC11513756; doi:10.1007/s10120-024-01546-x)
Supplement: Supplementary file 3 — Supplementary file3 (DOCX 25 KB) [file 10120_2024_1546_MOESM3_ESM.docx]

Online resource 2. Patients' characteristics with large type 3 tumor by histologic phenotypes

|  | Undifferentiated  large type 3  (n = 55) | Differentiated  large type 3  (n = 20) | *P* value |
| --- | --- | --- | --- |
| Sex |  |  | 0.5584 |
| Male | 37 (67%) | 12 (60%) |  |
| Female | 18 (33%) | 8 (40%) |  |
| Age, years |  |  |  |
| Mean ± SD | 71.2 ± 10.5 | 73.8 ± 9.4 | 0.3459 |
| Severe comorbidities |  |  | 0.0953 |
| Present | 13 (24%) | 1 (5%) |  |
| Absent | 42 (76%) | 19 (95%) |  |
| ECOG-PS |  |  | 0.3251 |
| 0 | 42 (76%) | 13 (65%) |  |
| ≥1 | 13 (24%) | 7 (35%) |  |
| Tumor location |  |  | 0.5107 |
| Upper | 13 (24%) | 6 (30%) |  |
| Middle | 12 (22%) | 2 (10%) |  |
| Lower | 20 (36%) | 6 (30%) |  |
| Whole | 10 (18%) | 6 (30%) |  |
| Tumor size, mm |  |  |  |
| Mean ± SD | 100 ± 22 | 94 ± 17 | 0.2629 |
| Neoadjuvant chemotherapy |  |  | 0.5344 |
| Yes | 13 (23%) | 3 (15%) |  |
| No | 42 (76%) | 17 (85%) |  |
| Type of gastrectomy |  |  | 0.5941 |
| Non-total gastrectomy | 21 (38%) | 6 (30%) |  |
| Total gastrectomy | 34 (62%) | 14 (70%) |  |
| pStage (JGCA 15th) |  |  | 0.1258 |
| IIA | 5 (9%) | 6 (30%) |  |
| IIB | 8 (14%) | 1 (5%) |  |
| IIIA | 13 (24%) | 7 (35%) |  |
| IIIB | 14 (25%) | 3 (15%) |  |
| IIIC | 15 (27%) | 3 (15%) |  |
| Adjuvant chemotherapy |  |  | 0.686 |
| Yes | 35 (64%) | 14 (70%) |  |
| No | 20 (36%) | 6 (30%) |  |

SD, standard deviation; BMI, body mass index; ECOG-PS, Eastern Cooperative Oncology Group Performance Status; JGCA, Japanese Gastric Cancer Association.
